# In Silico Modeling of Indigo and Tyrian Purple Single-Electron Nano-Transistors Using Density Functional Theory Approach

**DOI:** 10.1186/s11671-017-2193-7

**Published:** 2017-07-05

**Authors:** Sergey Shityakov, Norbert Roewer, Carola Förster, Jens-Albert Broscheit

**Affiliations:** 1 Department of Anesthesia and Critical Care, University of Würzburg, 97080 Würzburg, Germany; 2 Sapiotec Ltd., 97078 Würzburg, Germany

**Keywords:** Indigo, Tyrian purple, Single-electron transistor, Density functional theory

## Abstract

**Abstract:**

The purpose of this study was to develop and implement an in silico model of indigoid-based single-electron transistor (SET) nanodevices, which consist of indigoid molecules from natural dye weakly coupled to gold electrodes that function in a Coulomb blockade regime. The electronic properties of the indigoid molecules were investigated using the optimized density-functional theory (DFT) with a continuum model. Higher electron transport characteristics were determined for Tyrian purple, consistent with experimentally derived data. Overall, these results can be used to correctly predict and emphasize the electron transport functions of organic SETs, demonstrating their potential for sustainable nanoelectronics comprising the biodegradable and biocompatible materials.

**Graphical Abstract:**

In silico model and gate coupling of indigoid single-electron nano-transistors
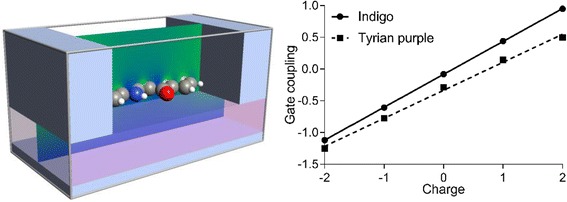

**Electronic supplementary material:**

The online version of this article (doi:10.1186/s11671-017-2193-7) contains supplementary material, which is available to authorized users.

## Background

The development of organic semiconductors that are capable of both hole and electron transport is of pivotal importance in designing new organic field-effect transistors (OFET) and optoelectronic nanodevices [1, 2]. Recently, the use of indigoids has been widely explored in such devices with a view to the creation of natural and sustainable semiconductors [3–5]. These botanical substances were derived from the plants *Indigofera tinctoria* and *Isatis tintora* and have been employed as organic dyes for the textile industry [6].

Indigoids, including indigo and its derivative, Tyrian purple, are insoluble agents with a very high melting point, a property which is explained by the presence of inter- and intramolecular stabilizing hydrogen bonds (H-bonds). On the other hand, the existence of π-skeleton intermolecular interactions also strongly influences the charge transport within indigo thin films [5]. Both materials are stable in terms of degradation within aerated conditions [7] and tend to form tiny and highly crystalline films upon evaporation, which exhibit promising charge transport properties [4]. Recent studies have also reported the development of indigo-based OLEDs (organic light-emitting diodes) and OFET devices due to the visible and near-infrared absorption spectra and electron transport characteristics of indigoid molecules [8, 9]. In particular, indigo and Tyrian purple show high and almost perfectly balanced electron and hole charge transport in OFET, owing to their reversible two-electron reduction and oxidation [8]. Another example of OLED devices contained a metal of 5-hydroxy-quinoxaline as a host component and indigo-based electroluminescent layer as a dopant material, comprising of a bisphenyl-squarilium compound [10].

Various computational approaches have been adapted and successfully implemented in the modeling of coherent transport in different types of molecular junctions, including density-functional theory (DFT), in combination with non-equilibrium Green’s functions or semi-empirical methods [11–14]. However, none of these in silico methods are applicable in the case of molecular single-electron transistors (SET) with incoherent electron transport [15]. Therefore, Kaasbjerg and Flensberg have devised a novel methodology, where they introduced a semi-empirical model for simulating the properties of molecular SETs, including a renormalization of the molecular charge states due to the environment polarization [16]. However, the possibility of indigoid integration within SET nanodevices remains to be investigated.

In the present work, to improve the performance of organic semiconductors, we modeled indigoid-based molecular SETs, which consist of the natural dye molecule weakly coupled to gold electrodes using the optimized DFT approach. These SET systems operate in the incoherent transport regime, and the electron transport is described by sequential tunneling of single electrons and a sequential transport mechanism, such as Coulomb blockade, rather than coherent, ballistic tunneling. The proposed in silico approach has the potential to correctly predict experimentally determined parameters and to explore the electronic properties of indigoids as bio-inspired materials for the development of novel organic semiconductors.

## Methods

**Fig. 1:**
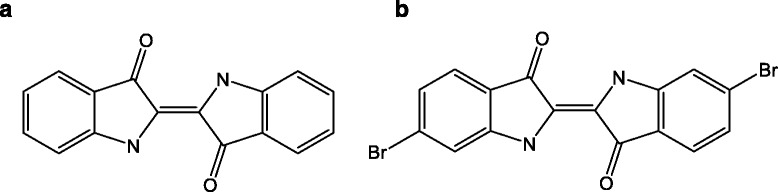
The molecular structure of *indigo* (**a**) and its derivative (6,6′-dibromoindigo) as *Tyrian purple* (**b**)

The molecular structures (Fig. 1a, b) of indigo (CID 5318432) and Tyrian purple (CID 5491378) were obtained from the PubChem database [17].

The molecular geometries were minimized using the steepest descent minimization algorithm, and the 3D sdf files were converted to the xyz coordinates by the Avogadro software [18]. The total partial charge (σ and π) distribution for the converted 3D structures (Additional file 1: Figure S1) was calculated by the MarvinSketch algorithm [19]. The SET microenvironment, consisting of the source, drain, and gate electrodes was built by the Virtual NanoLab v.2016.1 suite [20]. All the DFT calculations were performed using the Atomistix ToolKit software [20]. In particular, the LDA (local-density approximation) exchange correlation [21] was set with 75 Ha of mesh cut-off and 300 K of electron temperature. The energy zero parameter was measured as absolute energy for the SET molecular energy spectrum. For the SET nanodevice, the multi-grid Poisson solver was set to Neumann boundary conditions instead of multipole used only for the isolated molecules in the gas phase. The energy balance in the weak coupling regime was calculated from the *E* ^*island*^ *(N)* function, which determined the energy of the island as a function of the number of electrons in the island. Similar energy functions were introduced in the source and drain electrodes, *E* ^*source*^ *(N)* and *E* ^*drain*^ *(N)*. For the electron to move from the source electrode onto the island, the electron must have a lower energy on the island, such as the following:

$$ {E}^{source}(M)+{E}^{island}(N)\ge {E}^{source}\left(M-1\right)+{E}^{island}\left(N+1\right) $$

where *N* is the initial number of electrons on the island and *M* is the initial number of electrons in the source electrode. In order to move the electron from the island to the drain electrode, it should possess a lower energy in the drain electrode:

$$ {E}^{drain}(K)+{E}^{island}\left(N+1\right)\ge {E}^{drain}\left(K+1\right)+{E}^{island}(N) $$

where *K* is the initial number of electrons in the drain electrode.

Assuming that the electron with the maximum energy tunnels onto the island, then the maximum energy (*E* ^*source*^(*M*) − *E* ^*source*^(*M* − 1)) of the electron in the source electrode is described as follows:

$$ {E}^{source}(M)-{E}^{source}\left(M-1\right) = -W+\frac{eV}{2} $$

where *W* is the work function of the electrode and the *V* applied bias.

The above tunneling criterion, gives rise to the following condition:

$$ -W+\frac{eV}{2}+{E}^{island}(N)\ge {E}^{island}\left(N+1\right) $$

Similarly, $-W-\frac{eV}{2}$ is the minimum energy of an electron in the drain electrode, namely

$$ {E}^{island}\left(N+1\right)\ge -W-\frac{eV}{2}+{E}^{island}(N) $$

The requirement for a current in the device is therefore

$$ \frac{e\left|V\right|}{2}\ge \varDelta {E}^{island}(N)+W\ge -\frac{e\left|V\right|}{2} $$

where *ΔE* ^*island*^(*N*) = *E* ^*island*^(*N* + 1) − *E* ^*island*^(*N*) is the charging energy of the island.

## Results and Discussion

The configuration of modeled organic SETs was adjusted where the “indigoid” island only weakly coupled to the metal electrodes, tunneling the electrons from the source to the gate in a time-dependent manner. Therefore, to model this situation correctly, the DFT with LDA and a double-ζ-polarized basis set were applied [22]. The subsequent tunneling process from the island to the drain electrode could be in this regard referred to a transport mechanism, which is independent of the tunneling process into the island [23]. One of the prerequisites for indigoid molecules to be exploited in SET nanodevices is the presence of many alternating double and single bonds because such patterns delocalize the molecular orbitals, making it possible for electrons to move freely over the conjugated area [24].

**Fig. 2:**
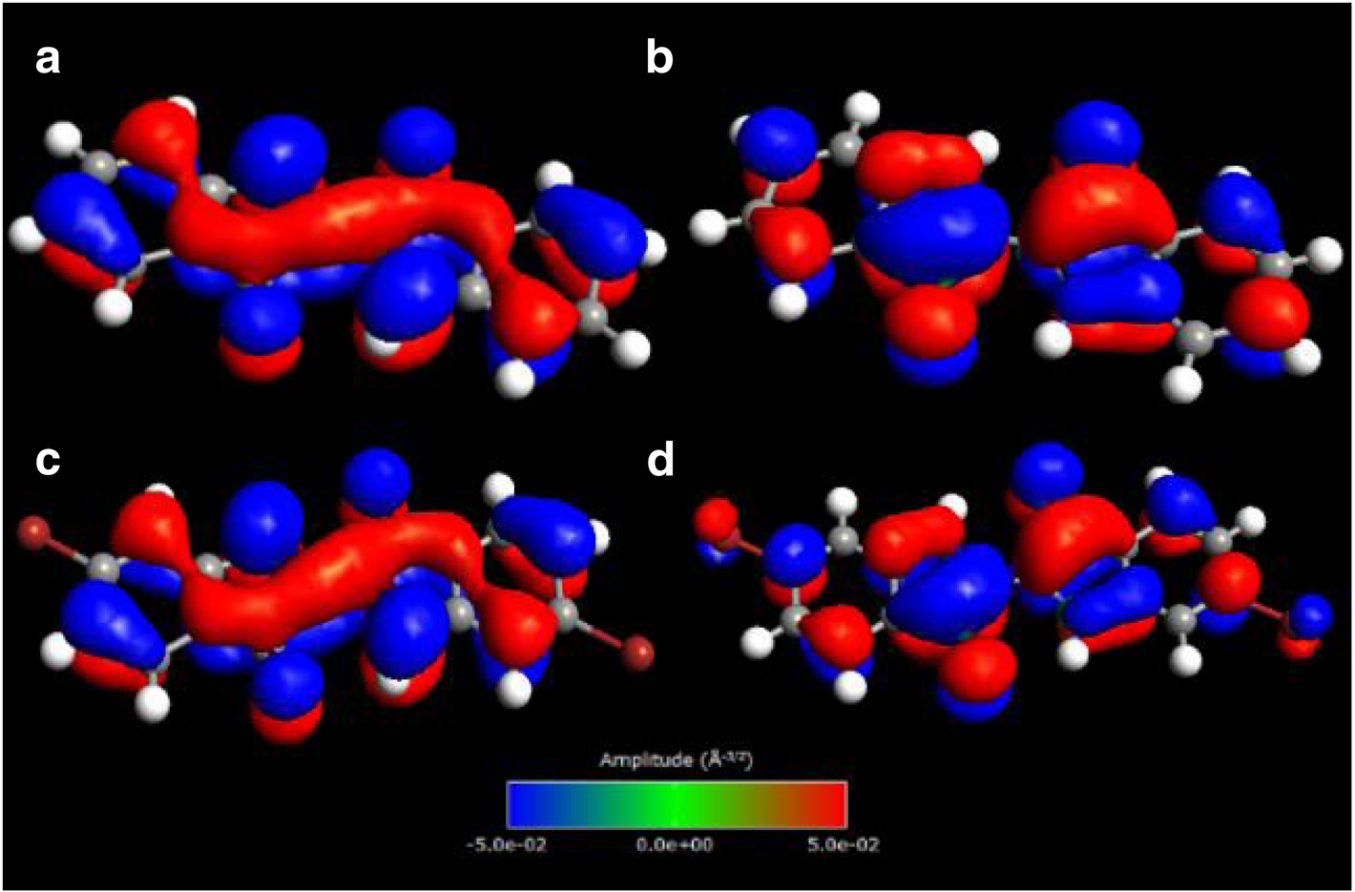
Isosurface plots of HOMO-LUMO eigenstates for indigo (**a**, **b**) and Tyrian purple (**c**, **d**) molecules. The molecular geometries were obtained by relaxing the molecules in the neutral state

After the convergence of DFT calculations, the neutral form (*q* = 0) of the indigoid molecules was inspected to determine the highest occupied (HOMO) and lowest unoccupied (LUMO) molecular orbital levels and to compare these values with the ionization (*E* ^*I*^) and affinity (*E* ^*A*^) energies. The occupations showed a level of 47 for HOMO (*E*_*HOMO*_ = *–*4.28 eV) and 48 for LUMO (*E*_*LUMO*_ = *–*2.94 eV) for indigo (Fig. 2a, b) and a level of 53 for HOMO (*E*_*HOMO*_ = *–*4.59 eV) and 54 for LUMO (*E*_*LUMO*_ = –3.21 eV) for Tyrian purple (Fig. 2c, d). The predicted values are in good agreement with the reported HOMO-LUMO energy gap of 1.3 eV for pure indigo previously published by Ramachandran and coauthor [9]. However, the LUMO level of Tyrian purple is deeper at –3.21 eV, which gives the ability to operate stably in the air for both p- and n-channels for at least 30 min with repeated cycling [4]. On the contrary, no air-stable n-type operation for indigo was observed in the experiment, probably due to the high-lying LUMO level (*–*2.94 eV) and oxygen atoms contribution as electron acceptors [4, 25].

To perform the evaluation of *E* ^*I*^ and *E* ^*A*^ for indigoids, an explicit calculation of the total energy (*E* ^*q*^) of the system with charge (*q)* was achieved. For this purpose, *E* ^*I*^ was defined as follows:

$$ {E}^I=E(N)-E\left(N-1\right)={E}^0-{E}^{+1} $$

where *E* ^0^ is the energy of the neutral system with *N* electrons and *E* ^+ 1^ is the energy of the positive ion with *N* − 1 electrons. Similarly, the affinity energy is given by the formula

$$ {E}^A=E(N)-E\left(N+1\right)={E}^0-{E}^{-1} $$

In general, the *E* ^*I*^ value is the cost of removing a single electron from the molecule that equals to HOMO, and the *E* ^*A*^ value is the energy gain of adding a single electron that corresponds to LUMO. However, the experimentally determined [4] values (*E*_*HOMO*_ = *–*5.5 and *E*_*LUMO*_ = *–*3.8 eV) for indigo and Tyrian purple (*E*_*HOMO*_ = *–*5.8 and *E*_*LUMO*_ = *–*4.0 eV) in the gas phase showed rather poor correlation with the calculated ones (Table 1), which must to be improved by the self-consistent calculations of these molecules in the SET environment.

**Table 1:** Charging energy *E* ^*N–1*^–*E* ^*N*^ of different charge states of indigo and Tyrian purple molecules in the gas phase

State	Charging energy (eV)	
+2	*−*10.69	*−*10.43
+1	*−*6.49^a^	*−*6.63^a^
0	–0.94(0.94)^b^	–1.28(1.28)^b^
*−*1	3.27	2.71

^a^ *E* ^*I*^

^b^ *E* ^*A*^

**Fig. 3:**
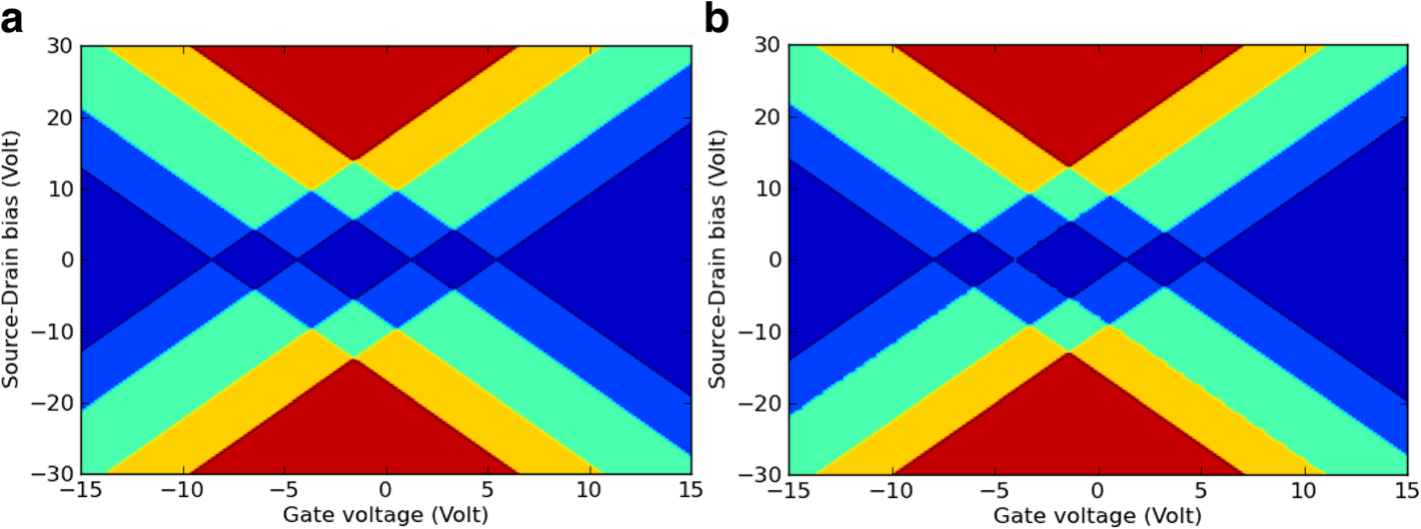
The charge stability diagram for *indigo* (**a**) and *Tyrian purple* (**b**) SETs, calculated in the gas phase. The *colors* show the number of charge states in the bias window for a given gate voltage and exhibit the typical diamond shapes also seen in experimental Coulomb blockade measurements. The color map is *blue* (0), *light blue* (1), *green* (2), *orange* (3), and *red* (4)

Next, the roughly estimated charge stability diagrams for indigo and Tyrian purple were calculated and plotted (Fig. 3), assuming that these molecules retain their properties in the gas phase even when it is part of the complete SET geometry. However, this model is only a rough approximation, since the molecular charge states are renormalized in the electrostatic SET environment. Therefore, the in silico model of these molecules connected to gold electrodes was implemented to investigate the electronic function of indigoids in a SET setup based on the calculated total energies for indigo and Tyrian purple in the gas phase. This preliminary test was performed through the formalism described in the M&M section as the energy balance in the weak coupling regime using the work function (*W* = 5.28 eV) of gold [26].

Furthermore, the dependence of the additional energies of the gate voltage (*V*_*G*_ *)* is mandatory in order to assess the linear coefficient, which is called the gate coupling constant (*α*).

$$ \varDelta E\left(N,{V}_G\right)=\varDelta E(N)+\alpha {V}_G $$

In order to simplify the simulated approach, the value *α* was set to 1, and the conditions for transmission through given molecular charge states were calculated by the following equation:

$$ -\frac{\left|{V}_{SD}\right|}{2}\le \varDelta E\left(N{V}_G\right)+W\le \frac{\left|{V}_{SD}\right|}{2} $$

It is clear from Fig. 3 that the conductance is directly related to the number of energy levels inside the bias window. In this regard, for any given value of *V*_*SD*_ (source-drain bias voltage) and *V*_*G*_, the calculated number of charge states in the bias window corresponded to the charge stability diagrams for indigo (Fig. 3a) and Tyrian purple (Fig. 3b). If the charging energy of the “indigoid” island in the gas phase is modified by an electrostatic gate through a tuning of the *V*_*G*_ parameter, the energy levels of SET are moved in and out of the bias window.

**Fig. 4:**
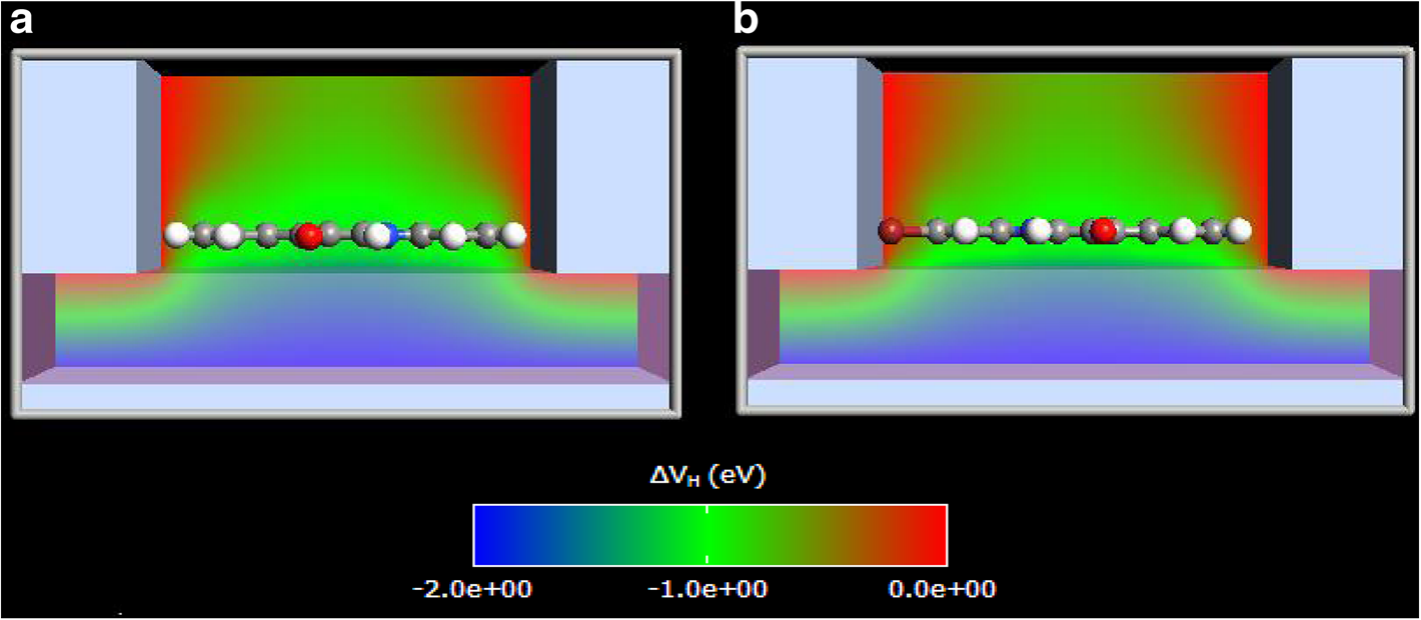
The geometry of the molecular SET environment of indigo (**a**) or Tyrian purple (**b**). Both molecules are modeled within an electrostatic environment, which simulates metal source-drain electrodes on top of a dielectric substrate (shellac) with a metal back-gate. ΔV_H_ is denoted for the Hartree potential difference calculated by solving the Poisson equation. The *contour plot* shows the induced electrostatic potential for a *V*_*G*_ value of 2 V and zero source-drain bias. The molecular geometries were obtained by relaxing the molecules in the neutral state

In order to improve accuracy, the indigoid molecules were positioned in a SET environment, which consists of the source, drain, and gate gold electrodes. The system was set up for the DFT analysis, where the organic molecule was placed on top of a 3.7 Å thick dielectric slab, with a metal back-gate and surrounded by a source and drain metal electrodes. Since the organic molecules are only weakly coupled to the electrodes, the main interactions with the electrodes are electrostatic forces [27]. These interactions were modeled using a continuum model of the electrodes with shellac dielectric. The latter component, known as natural resin (*ε*_r_ = 3.9ε_0_), is widely used as a substrate and a dielectric layer for OFETs [28]. Finally, the whole system was modeled as an isolated device configuration due to the transport mechanism, which is a sequential tunneling, rather than a coherent ballistic tunneling (Fig. 4a, b).

**Fig. 5:**
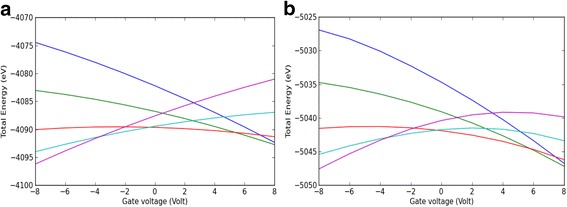
*E*(*q*, *V*_*G*_) as a function of *V*_*G*_ for *indigo* (**a**) and *Tyrian purple* (**b**) SET nanodevices. This function was calculated in the SET environment. *Different curves* represent different charge states of the natural dye molecule,* blue* (*−*2), *green* (*−*1), *red* (0), *turquoise* (1), and *violet* (2)

Considering that the gold electrodes function as electron reservoirs, the energy of an additional electron was described by the work function of these electrodes. Hence, the lowest energy was detected at the most stable and uncharged state of indigoid molecules for given *V*_*G*_ values, where negative *V*_*G*_ stabilized positively charged ions and vice versa (Fig. 5a, b).

On the contrary, the gate dependence is close to linear, and the slope is related to the charge state of the indigoid molecule. To investigate this dependence further, a relation describing the total energy as *E*(*q*, *V*_*G*_) and charge function was used [29]:

$$ E\left(q,{V}_G\right)=\alpha q{V}_G $$

The relationship between *E*(*q*, *V*_*G*_) and *V*_*G*_ is non-linear because the atoms closest to the dielectric region screen *V*_*G*_ for the rest of the molecule, decreasing the gate coupling. A difference in the charges on different atoms in the indigoid molecule alters a molecular dipole, which in turn contributes to *E*(*q*, *V*_*G*_).

In the regions starting from 0 to 5 gate voltage, the minimal *E*(*q*, *V*_*G*_) states were detected for Tyrian purple due to the bromination of this molecule (Fig. 5b). Since, the polarizability of a halogen atom increases in the order of F < Cl < Br, bromine shows large polarizability between the halogen atoms [30]. These data also reflected the experimentally determined relative permittivity values of 4.3 for indigo and 6.2 for Tyrian purple calculated from the geometric capacitance at high frequency (>1.0 MHz) [4]. Moreover, the indigoid-based nanodevices exhibited ambipolar operation with the electron (*μ*_*e*_) and hole (*μ*_*h*_) mobilities from 10^−2^ to 0.2 cm^2^(V × s)^−1^. In a more recent study, higher mobilities in Tyrian purple were also reported with the *μ*_*e*_ and *μ*_*h*_ values of 0.4 cm^2^(V × s)^−1^ [31]. However, the analyzed SET nanodevices are different from any conventional OFET, which only controls the charge density between the electrodes but on the single-electron transport through the energy modifications of molecular orbitals [32, 33].

**Fig. 6:**
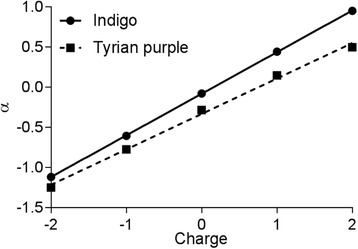
Linear relationship of the gate coupling (*α*) and the charge values for *indigo* and *Tyrian purple* SETs

Therefore, the gate coupling effect (Fig. 6) for Tyrian purple is represented by a lower value (*α* = 0.44) than that for indigo (*α* = 0.52) as a result of the weaker coupling of the indigo derivative to the metal gate. Hence, indigo is located closer to the shellac substrate to allow all atoms to be almost identically shifted by the gate potential, which shows a more linear relationship between the total energy and *V*_*G*_.

**Fig. 7:**
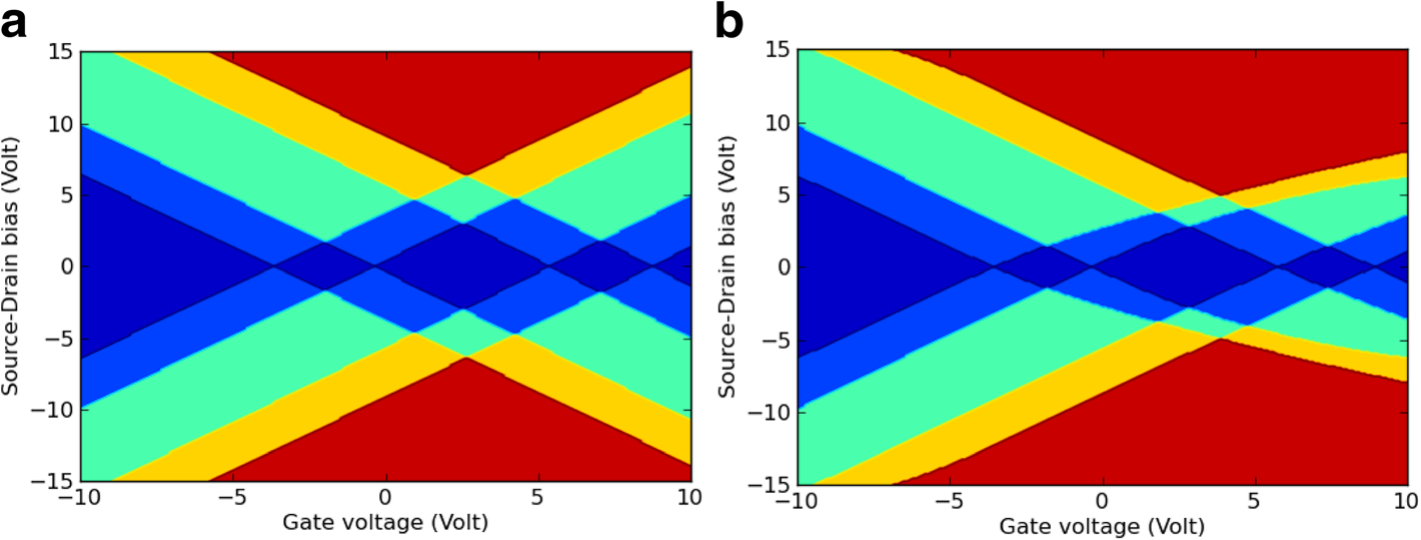
The full self-consistently calculated charge stability diagrams for *indigo* (**a**) and *Tyrian* *purple* (**b**) SETs. These diagrams were calculated for the SET environment. The *colors* show the number of charge states in the bias window for a given *V*_*G*_. The color map is* blue* (0), *light blue* (1), *green* (2), *orange* (3), and *red* (4)

Additionally, the refined *E* ^*I*^ and *E* ^*A*^ coefficients (Table 2) of indigo and Tyrian purple molecules and their corresponding charge stability diagrams (Fig. 7a, b) calculated in the SET environment showed a good correlation with the experimentally determined data previously described elsewhere [4]. The relatively high values of ionization energy and the low values of electron affinity for the indigoid compounds also make them useful for OLED applications owing to the low energy barrier for the creation of holes and the injection of electrons [9].

**Table 2:** Charging energy *E* ^*N–1*^–*E* ^*N*^ of different charge states of indigo and Tyrian purple molecules in the SET environment

State	Charging energy (eV)	
+2	*−*7.15	*−*6.63
+1	*−*5.46^a^	*−*5.43^a^
0	−2.42(2.42)^b^	−2.48(2.48)^b^
*−*1	*−*0.7	−0.89

^a^ *E* ^*I*^

^b^ *E* ^*A*^

From these diagrams, it is clear that the non-linear dependence of *E*(*q*, *V*_*G*_) on *V*_*G*_ is not detectable because the diagrams only depend on the *ΔE* values between the charge states. The excitation energy for indigo is greater than for Tyrian purple, but this energy term of the second electron is smaller than for the first one in both modeled systems. Overall, in terms of stability, mobility, low operating voltage, and ON/OFF ratio, the indigoid OFET nanodevices are among the best reported in the literature [34].

## Conclusions

SETs have been proposed as a future alternative to modern Si-based electronics. The use of single molecules, or nanoscale collections of single molecules, as electronic components is the ultimate goal for conventional electronics in order to minimize electrical circuits, replacing bulk materials. Some SETs were successfully realized within individual carbon nanotubes, organic molecules, and self-assembled gold nanoparticles in an experiment [35–37]. The other example of promising SETs based on graphene is being heavily investigated in research labs, because this material promises a band gap tunable by electrostatic gates [38]. For this reason, we have developed the in silico model of the indigoid-based SET nanodevices using the optimized DFT methodology. This theoretical approach was able to correctly predict the main electronic properties of the natural dye molecules weakly coupled to gold electrodes. The improved electron transport characteristics were determined for Tyrian purple SET system, operating in the incoherent transport regime and describing by sequential tunneling of single electrons and sequential transport mechanism, such as Coulomb blockade, rather than coherent, ballistic tunneling. As the best available organic semiconductors, indigoids demonstrate the potential for sustainable electronics based on the biodegradable and biocompatible materials. Concerning the aforementioned experimental data, our simulation results inspire confidence that indigoid-based SET devices will work and be competitive to normal transistors soon or more in the far future.

## Additional file

Additional file 1: Figure S1. Total partial charge distribution calculated for indigo (A) or Tyrian purple (B). Legend: Both molecules are protonated, depicted as ball-and-stick models, and colored according to their atomic composition (CPK). (PDF 164 kb)
