# Supplementary figures and images for: In Silico Modeling of Indigo and Tyrian Purple Single-Electron Nano-Transistors Using Density Functional Theory Approach
Source: Nanoscale Res Lett. 2017 Jul 5;12:439. doi: 10.1186/s11671-017-2193-7 (PMC5498432; doi:10.1186/s11671-017-2193-7)

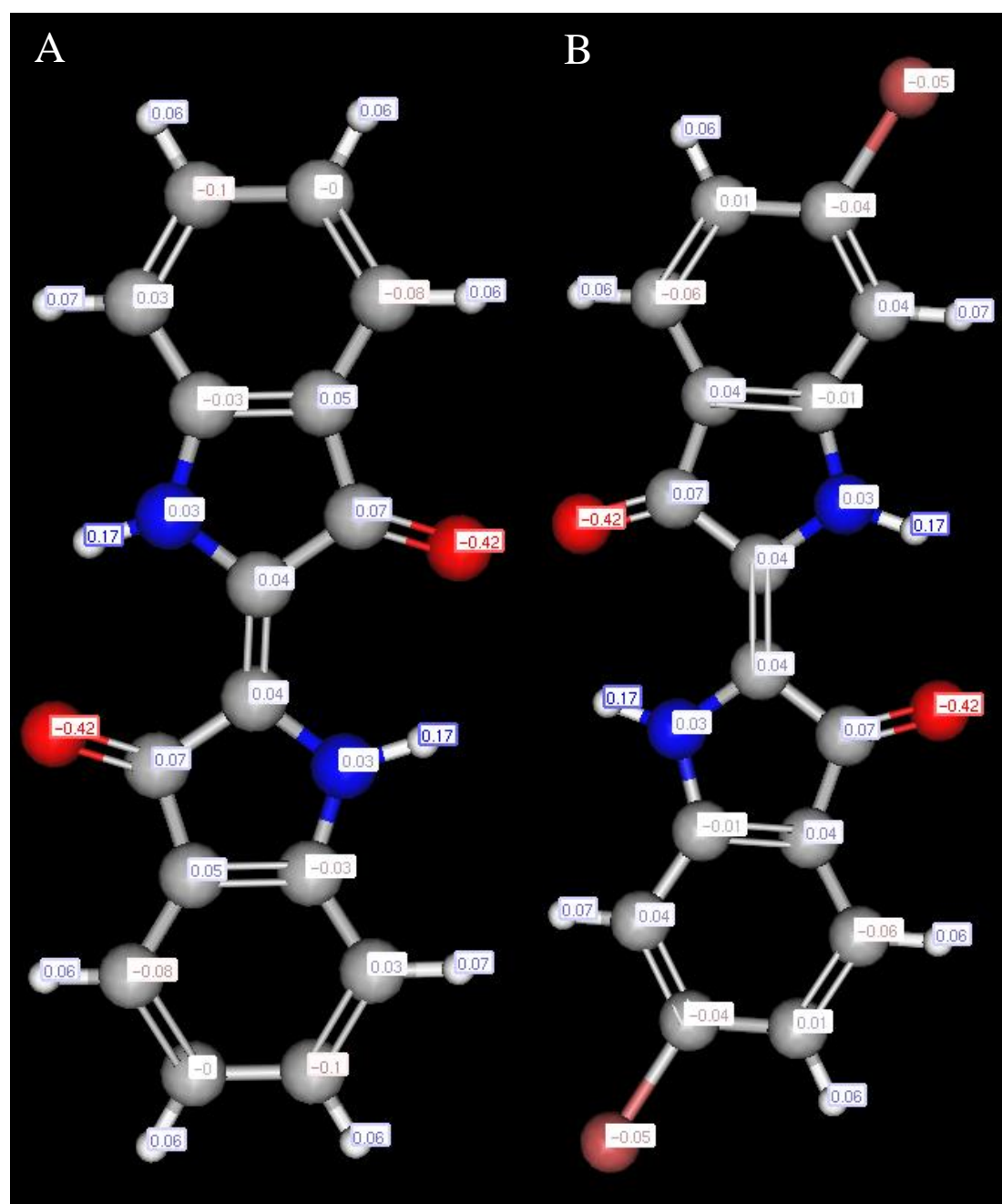

**Figure S1**

Supplement: Additional file 1: Figure S1. — Total partial charge distribution calculated for indigo (A) or Tyrian purple (B). Legend: Both molecules are protonated, depicted as ball-and-stick models, and colored according to their atomic composition (CPK). (PDF 164 kb) [file 11671_2017_2193_MOESM1_ESM.pdf]
